# Ultrasound imaging of dorsal neck muscles with speckle tracking analyses – the relationship between muscle deformation and force

**DOI:** 10.1038/s41598-019-49916-1

**Published:** 2019-09-23

**Authors:** Gunnel Peterson, Shaun O’ Leary, David Nilsson, Katherine Moodie, Kylie Tucker, Johan Trygg, Anneli Peolsson

**Affiliations:** 1 Centre for Clinical Research Sörmland, Uppsala University, Eskilstuna, Sweden; 2 Department of Medical and Health Sciences, Division of Physiotherapy, Faculty of Health Sciences, Linköping University, Linköping, Sweden; 3 School of Health and Rehabilitation Sciences, The University of Queensland, Brisbane, Australia; 4 Physiotherapy Department, Royal Brisbane and Women’s Hospital, Queensland Health, Queensland, Australia; 5 Computational Life Science Cluster (CLiC), Department of Chemistry, Umeå University, Umeå, Sweden; 6 The University of Queensland, School of Biomedical Sciences, Brisbane, Australia

**Keywords:** Skeletal muscle, Medical research

## Abstract

The development of methods of non-invasive measurement of neck muscle function remains a priority in the clinical sciences. In this study, dorsal neck muscle deformation vs time curves (deformation area) were evaluated against incremental force, recorded from non-invasive real-time ultrasound measurement. The results revealed subject-specific moderate to strong linear or non-linear relationships between deformation and force. Test-retest variability showed strong reliability for all five neck muscles summed together and fair to good reliability for the five muscles evaluated separately. Multivariate statistics were used to analyse the interactions between the dorsal neck muscles during different percentages of maximal voluntary contraction (MVC). Low force (10–20% MVC) was related to muscle shortening; higher force (40–80% MVC) showed combination of shortening and elongation deformation in the muscle interactions. The muscle interactions during isometric MVC test were subject-specific, with different combinations and deformations of the five neck muscles. Force ≥40% MVC were associated with a forward movement of the cervical spine that affected the ultrasound measurement of the dorsal neck muscles. Ultrasound with speckle-tracking analyses may be best used to detect low levels (<40% MVC) of neck muscle activity.

## Introduction

The neck muscle function is complex^1^, dysfunction of superficial and deep muscle layers are related to persistent neck pain^2–5^. The understanding of neck muscle function has improved^1,5–8^, but the challenge has been to develop methods to evaluate impairment in neck muscles and evaluate results of exercise interventions that can be used in research and clinical practice.

The most common method used in research is electromyography (EMG). Surface EMG and invasive fine wire EMG have shown altered dorsal neck muscle behaviour in the presence of neck pain^5,9^. However, invasive EMG can be uncomfortable and is not applicable in the clinical setting. In contrast, real-time ultrasound imaging can non-invasively detect and quantify skeletal muscle deformation providing information regarding local changes within the muscle during activity^10,11^. Speckle tracking is a method that provides information on muscle deformation; i.e., elongation and shortening of the muscle^12–15^. This method provides the ability to investigate both superficial and deep muscle layers simultaneously^10,12,13^ and may improve diagnostics in neck pain and evaluate exercise regimens. Moreover, ultrasound investigation is a relatively low-cost technique, with no known risks or contraindications. Lopata *et al*.^11^ showed that a strain estimation method facilitated the detection of tissue motion by calculation deformation curves as a function of time. They measured local deformations in two directions simultaneously in the biceps brachii muscle in response to force, during both electrical stimulation and voluntary contraction. Studies have shown altered neck muscle deformation curves in individuals with whiplash-associated disorder (WAD) compared to healthy individuals^12,13,16^, using a speckle-tracking method calculating deformation vs time curves (area under the curve) that has the capacity to distinguish between elongation and shortening deformation. However, the reliability and validity against force have not been adequately investigated. A previous study conducted by our team showed that muscle deformation during isometric contractions of the cervical extensor muscles performed over a range of intensities were significantly related to torque, however the relationship was weak (r^2^ = 0.03–0.18)^17^. This previous study however, utilized the average root mean square (RMS) values of deformation curves. We postulate that this method may not optimally identify deformation. Instead, we propose that cervical muscle deformation versus time curves would provide a more accurate method for evaluating ultrasound speckle tracking images that would be more strongly related to changes in force. Additionally, the relationship between deformation and isometric force, and specifically the interactions between muscles and isometric force, can be investigated by means of multivariate data analysis. In general, multivariate techniques are beneficiary to use for analysis of many variables^18^ to summarize the content in a few components or latent variables and also to find the relationship to a response variable, e.g. the isometric force.

The aim of this study was to further evaluate a method of quantifying deformation in five dorsal neck muscles by calculation of muscle deformation vs time curves (area under the curve). It was hypothesised that the deformation vs time curves methods would more accurately reflect isometric cervical extension muscle force than that shown previously^17^ using the average root mean square (RMS) method. The relationship between deformation and isometric force was evaluated, as well as the interaction between the neck muscles and isometric force. A second aim was to investigate test-rest reliability among three tests.

## Results

The raw values for the deformation areas (before normalization) are presented in Table 1.

**Table 1:** Deformation values for the five neck muscles.

MVC		10%	20%	40%	60%	80%	100%
**Test time**		8.0 (0.65)	8.0 (0.58)	8.4 (1,1)	8.1 (0.57)	7.9 (0.82)	7.3 (0.84)
**Total area**	TR	19.2 (12.9)	25.4 (8.6)	40.0 (33.1)	58.6 (48.3)	56.1 (46.8)	63.6 (46.5)
	SP	30.7 (22.8)	35.1 (17.1)	55.8 (32.3)	74.3 (26.4)	82.6 (39.1)	159.1 (131.6)
	Scap	47.5 (32.89	54.8 (28.7)	93.3 (77.1)	112.3 (63.0)	130.0 (87.3)	152.9 (112.2)
	Scerv	57.3 (28.7)	72.3 (34.6)	116.9 (60.4)	123.6 (103.9)	131.9 (88.8)	123.1 (94.5)
	MF	35.8 (21.8)	61.4 (27.8)	92.6 (41.4)	116.9 (74.9)	111.8 (84.2)	124.9 (85.6)
**Elongation**	TR	4.1 (3.9)	10.4 (7.1)	9.2 (11.8)	24.6 (26.4)	34.2 (49.0)	30.6 (35.4)
	SP	10.1 (14.0)	12.7 (8.4)	25.9 (24.3)	39.4 (36.7)	66.0 (47.2)	149.3 (136.6)
	Scap	22.1 (36.3)	27.8 (32.6)	57.0 (62.8)	73.9 (55.6)	94.3 (99.5)	145.1 (119.7)
	Scerv	25.6 (20.7)	52.1 (42.8)	76.4 (48.5)	79.7 (56.3)	116.5 (94.4)	115.7 (100.3)
	MF	18.1 (22.9)	44.9 (33.8)	76.6 (50.1)	94.4 (73.2)	93.1 (88.5)	111.8 (93.9)
**Shortening**	TR	15.2 (14.9)	14.1 (10.8	25.0 (23.7)	24.8 (23.6)	21.8 (21.4)	32.9 (53.0)
	SP	20.6 (24.9)	20.7 (18.3)	29.7 (29.5)	32.1 (31.7)	16.6 (18.9)	9.8 (38.0)
	Scap	25.4 (19.9)	28.2 (25.1)	24.8 (27.1)	27.5 (35.6)	35.7 (44.4)	7.8 (14.1)
	Scerv	31.7 (37.0)	21.8 (31.2)	29.4 (45.4)	23.4 (44.6)	15.4 (18.1)	7.3 (12.2)
	MF	17.6 (17.1)	16.7 (17.4)	16.7 (18.9)	13.5 (18.0)	18.7 (21.4)	13.0 (22.4)

Data are mean ± SD. Muscle deformation area (% deformation) during percent of maximal voluntary contraction in the five dorsal neck muscles. Abbreviations; TR, trapezius; SP, splenius capitis; Scap, semispinalis capitis; Scerv, semispinalis cervicis; MF, multifidus/rotatores; MVC, maximal voluntary contraction; 10%; 10 percent of MVC, 20%; 20 percent of MVC, 40%; 40 percent of MVC, 60%; 60 percent of MVC; 80%, 80 percent of MVC; 100%, maximal voluntary contraction.

### Relationship between total deformation area for the five neck muscles and force

**Figure 1:**
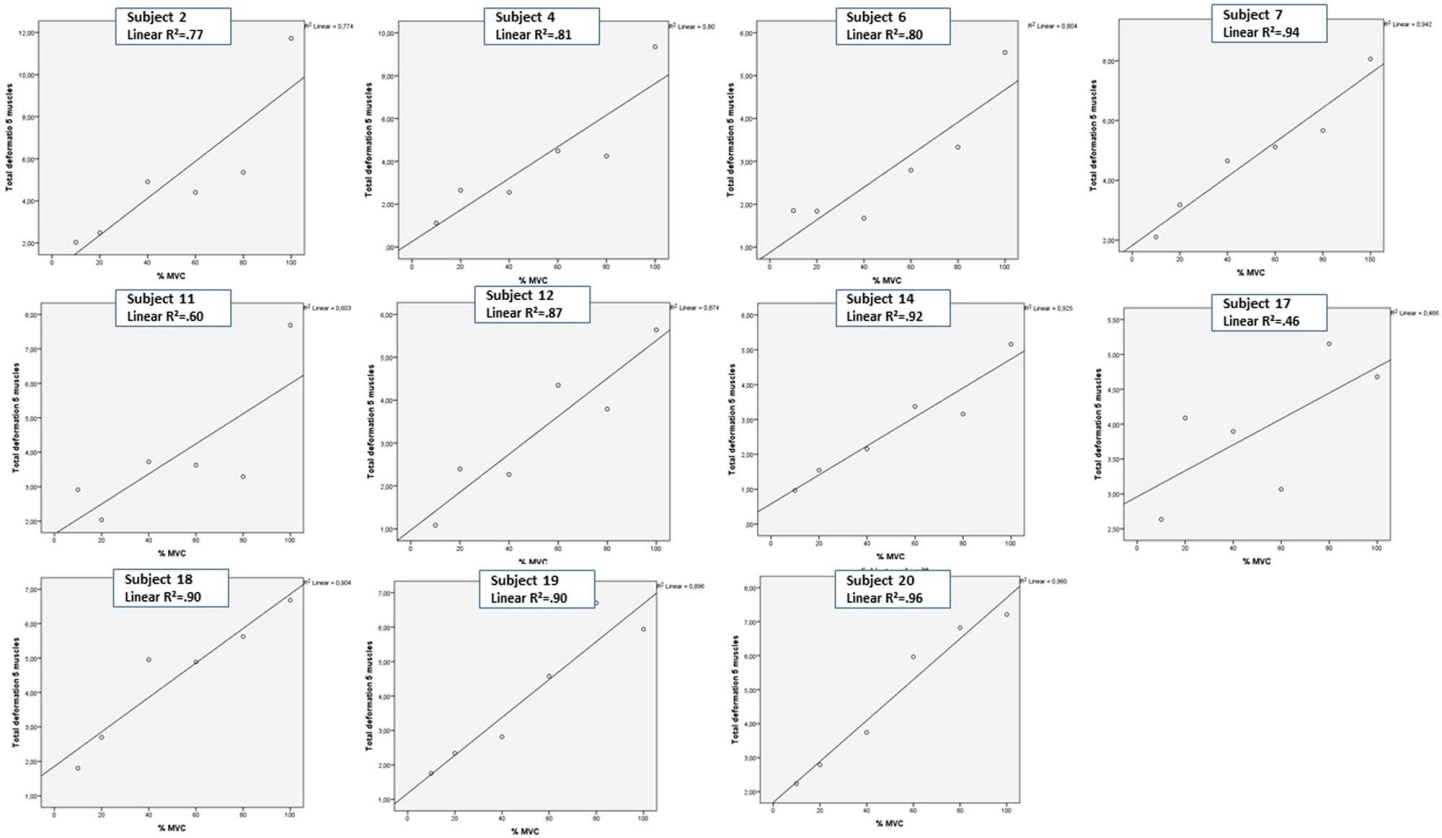
Subject specific relationship between deformation and force – linear relationship. The raw data mean deformation area under the curve for all five neck muscles summed is plotted against the percent of maximal voluntary contraction (%MVC) for each subject (% MVC). A visual inspection of linear and non-linear (quadratic) relationship showed that linear curves best fitted the data for eleven individuals. For two subjects (11 and 17) the relationship was moderate.

**Figure 2:**
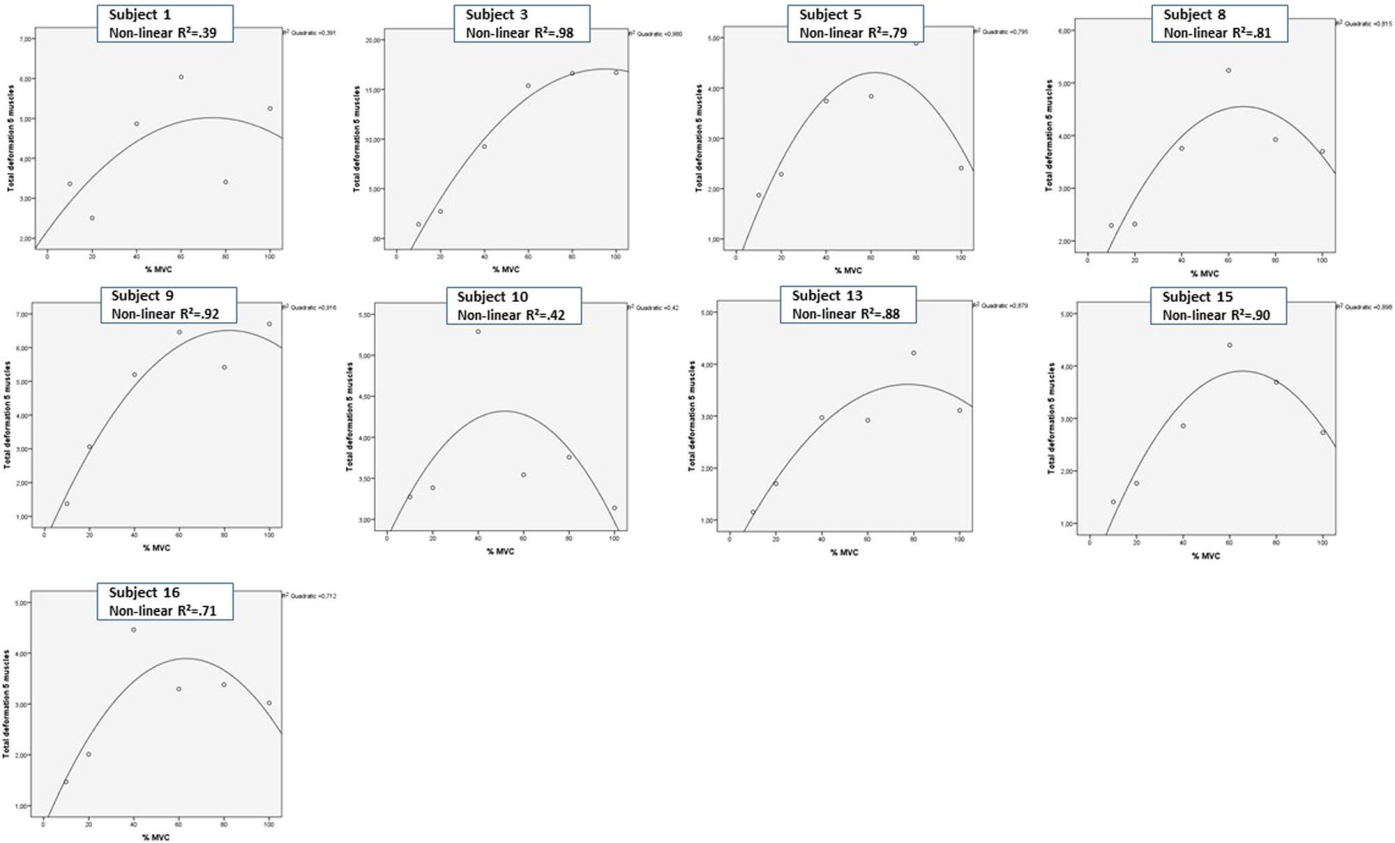
Subject specific relationship between deformation and force – non-linear relationship. The raw data mean deformation area under the curve for all five neck muscles summed is plotted against the percent of maximal voluntary contraction (% MVC) for each subject. For nine individuals, non-linear curves best fitted data, however for two individuals the relationship were moderate (1 and 10). The non-linear curves for six individuals (subject 3, 5, 9, 13, 15, 16) showed a linear relationship for deformation related to 10%, 20% and 40% MVC.

**Figure 3:**
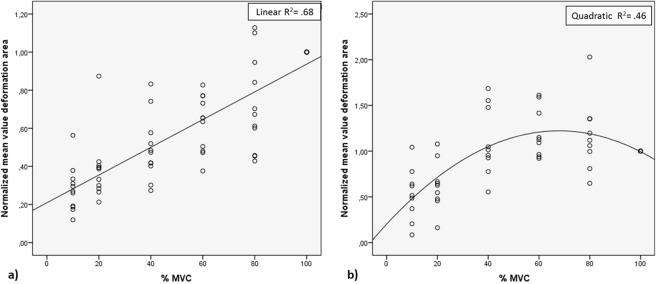
Relationship between deformation and force. The normalized mean deformation area under the curve for each subject is plotted against the percent of maximal voluntary contraction (%MVC). (**a**) A linear relationship is observed for all five neck muscles summed in some individuals (n = 11; R2 = 0.68). (**b**) A non-linear relationship is observed for all five neck muscles in other individuals (n = 9; R2 = 0.46).

The raw data analyses showed that eleven subjects, seven men and four women, displayed a linear relationship between the %MVC and the deformation area (Table 2 and Fig. 1). Two showed moderate (R^2^ = 0.46 and 0.60) and nine showed strong to very strong linear relationships (R^2^ = 0.77 to 0.96, Table 2). Nine subjects, five men and four women, displayed non-linear relationships between the %MVC and the deformation area. Two showed moderate (R^2^ = 0.39 and 0.42) and seven showed strong to very strong non-linear relationships (R^2^ = 0.71 to 0.98, Table 2 and Fig. 2). When analysed in groups, the association between the normalized deformation area and force was strong for those with a linear relationship (R^2^ = 0.68) and moderate for those with a non-linear relationship (R^2^ = 0.46) (Fig. 3).

**Table 2:** Each subjects (n = 20) adjusted R^2^ values (mean value for three tests) for linear and non-linear relationship between the deformation area for the five neck muscles (trapezius, splenius, semispinalis capitis, semispinalis cervicis and multifidus/rotatores) and percent of maximal voluntary contraction (MVC).

Subject	Gender	Linear	Subject	Gender	Non-linear
20	F	0.96	3	M	0.98
7	M	0.94	9	M	0.92
14	M	0.92	15	F	0.89
18	M	0.90	13	F	0.88
19	F	0.90	8	F	0.81
12	M	0.87	5	M	0.79
6	F	0.81	16	F	0.71
4	M	0.81	10	M	0.42
2	M	0.77	1	M	0.39
11	F	0.60			
17	M	0.46			

M; male, F; female.

### Relationship between the normalized deformation areas for five neck muscles and the percent of maximal voluntary contraction

When the normalized data for all subjects (n = 20) were combined, we found that, for all five muscles summed together, the mean normalized deformation values were significantly related to the %MVC: F (2.8, 53.6) = 29.2, p < 0.001. The individual neck muscles showed a significant relationship between the deformation values and the %MVC: the trapezius, F (1.4, 26.1) = 5.1, p < 0.03; the splenius, F (1.8, 35.1) = 9.5, p < 0.001; the semispinalis capitis, F (2.9, 56.6) = 3.5, p = 0.02; the semispinalis cervicis; F (1.8, 33.9) = 4.6, p = 0.02; and the multifidus muscles F (2.1, 40.5) = 6.1, p < 0.01.

### Multivariate analyses, two-way interactions between the five dorsal neck muscles

**Figure 4:**
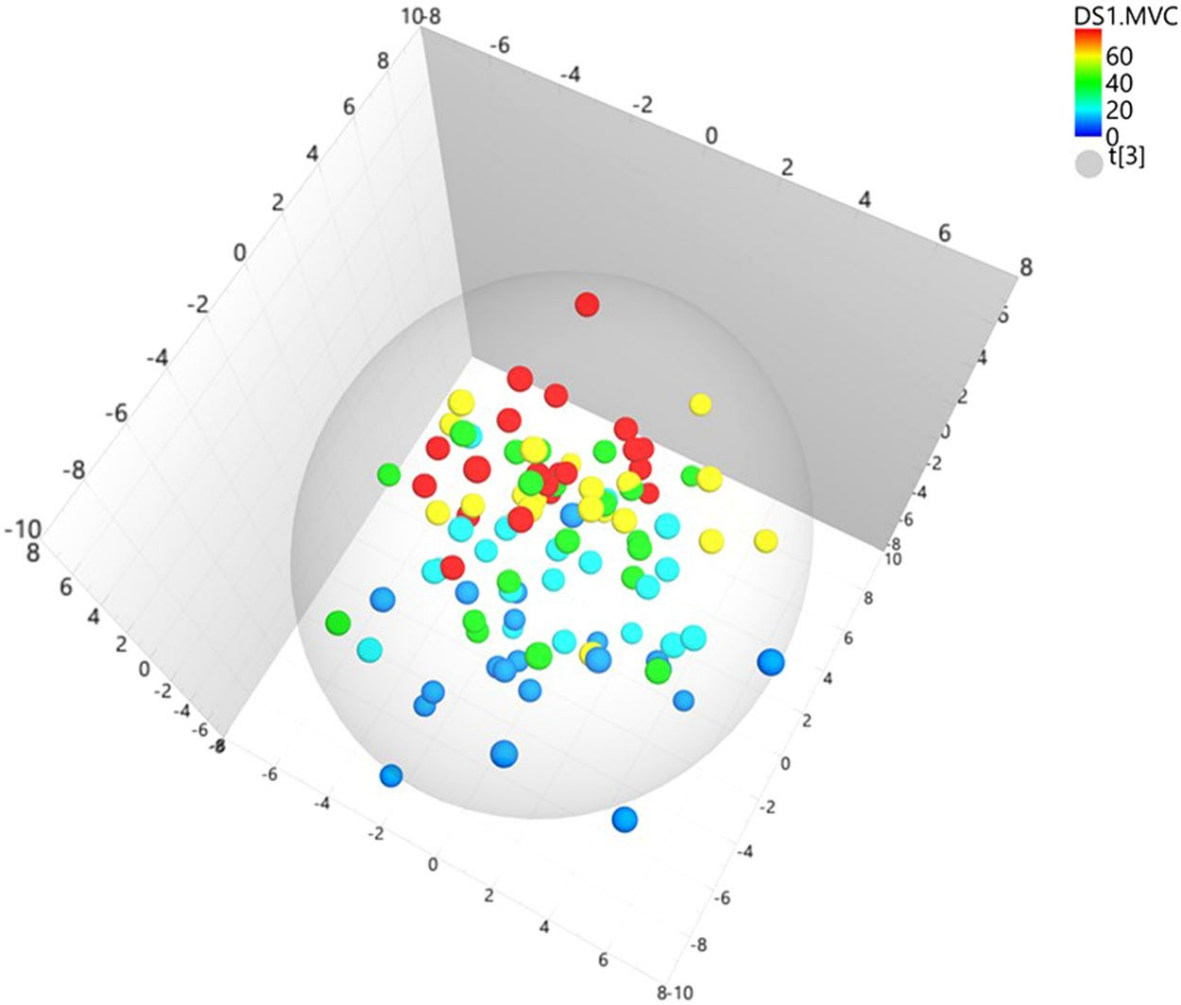
Principal component analysis (PCA). Score scatter plot for principal component analysis, PCA, created for the five dorsal muscles (TR, SP, Scap, Scerv, MF) and their two-way interaction terms between elongation and shortening areas. The PCA score plot displays systematic variations and patterns in large heterogeneous data sets. The first three components, explaining 24%, 15% and 13% respectively, reveals a trend from low to high maximum voluntary isometric contraction (MVC), (dark blue dots; 10% MVC, light blue dots; 20% MVC, green dots; 40% MVC, yellow dots; 60% MVC and red dots; 80% MVC). Each dot corresponds to one subject.

**Figure 5:**
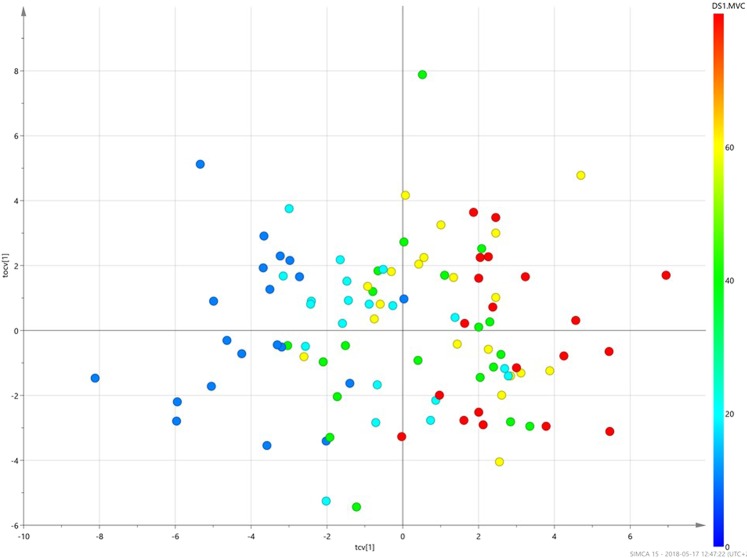
Orthogonal partial least squares analysis (OPLS) score scatter plots. Cross-validated score plot of orthogonal partial least squares regression, OPLS, between two-way dorsal muscle interactions, X, and maximum voluntary isometric contraction as response, y. Dark blue; 10% MVC, light blue; 20% MVC, green; 40% MVC, yellow; 60% MVC, red; 80% MVC. The two-component OPLS model with one predictive component along the x-axis and one orthogonal component along the y-axis had R2Y and Q2Y values of 0.61 and 0.55 respectively. Leave-one-subject-out cross-validation was carried out and the cumulative prediction outcome can be seen in the plot where one dot corresponds to one subject. A clear trend can be seen, with low % maximal voluntary contraction (MVC) values to the left and high % MVC values to the right.

Principal component analysis (PCA) of two-way dorsal muscle interactions generated a score scatter plot for the first three components explaining 52% of the variance in the data, (Fig. 4). The dorsal muscle interactions can be studied by calculating and analyzing all possible two-way interaction terms of the five dorsal muscles for each % MVC. No serious outliers were detected in the plot that displays a clear trend from low to high MVC. The data were further examined with orthogonal partial least (OPLS) supervised regression to assess the strength of relationships between the dorsal muscle interactions and the MVC. The fitted two-component OPLS model (one predictive and one orthogonal) had a model explained variance, R^2^Y, of 0.61 and a predictive model explained variance, Q2Y, of 0.55. The cross-validated OPLS scores of the predictive and the orthogonal components (Fig. 5) showed a clear trend from low MVC to high MVC trials. The lowest levels, 10 and 20%, are mostly separated from the two highest MVC levels, 60 and 80%. The observations of the intermediate level 40% MVC are scattered in the middle of the plot.

**Figure 6:**
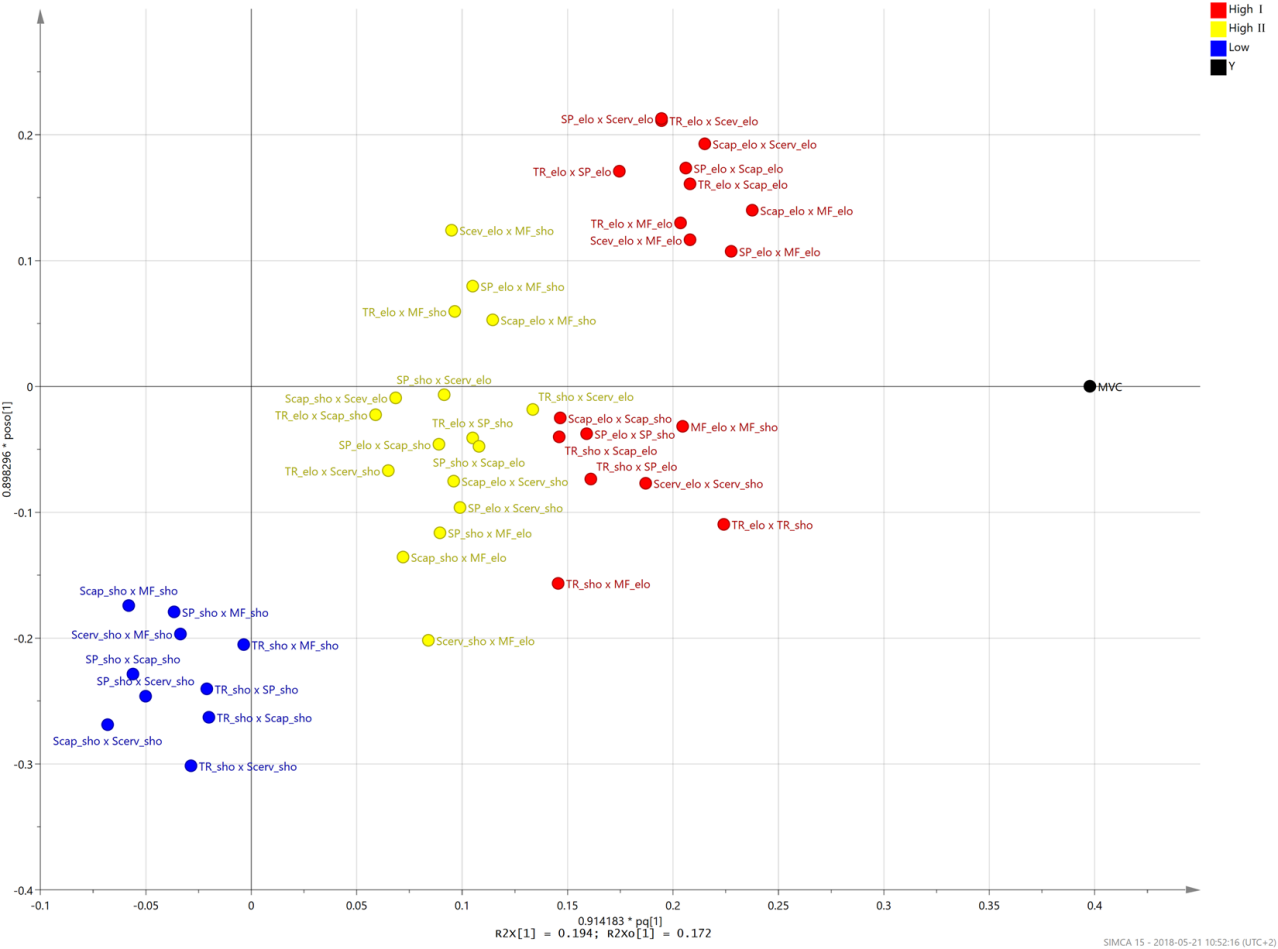
Orthogonal partial least squares (OPLS) loading. Loading plot from orthogonal partial least squares (OPLS) regression modelling of two-way dorsal muscle interactions and the maximum voluntary isometric contraction set as response. The plot displays the influence of the variables on the model and their correlation to MVC. The variables, i.e. the two-way dorsal muscle interaction terms, can be categorized into three types depending on their relationship to the response. The low/blue interactions are typically activated during low MVC values, i.e. 10 and 20%, and they are weak during higher MVC exercises. The High I/red interactions are typically activated during high MVC, i.e. 60 and 80% and they are important for the model. The High II/yellow interactions are also activated during higher MVC, but they are less pronounced than the High I/red interactions.

**Figure 7:**
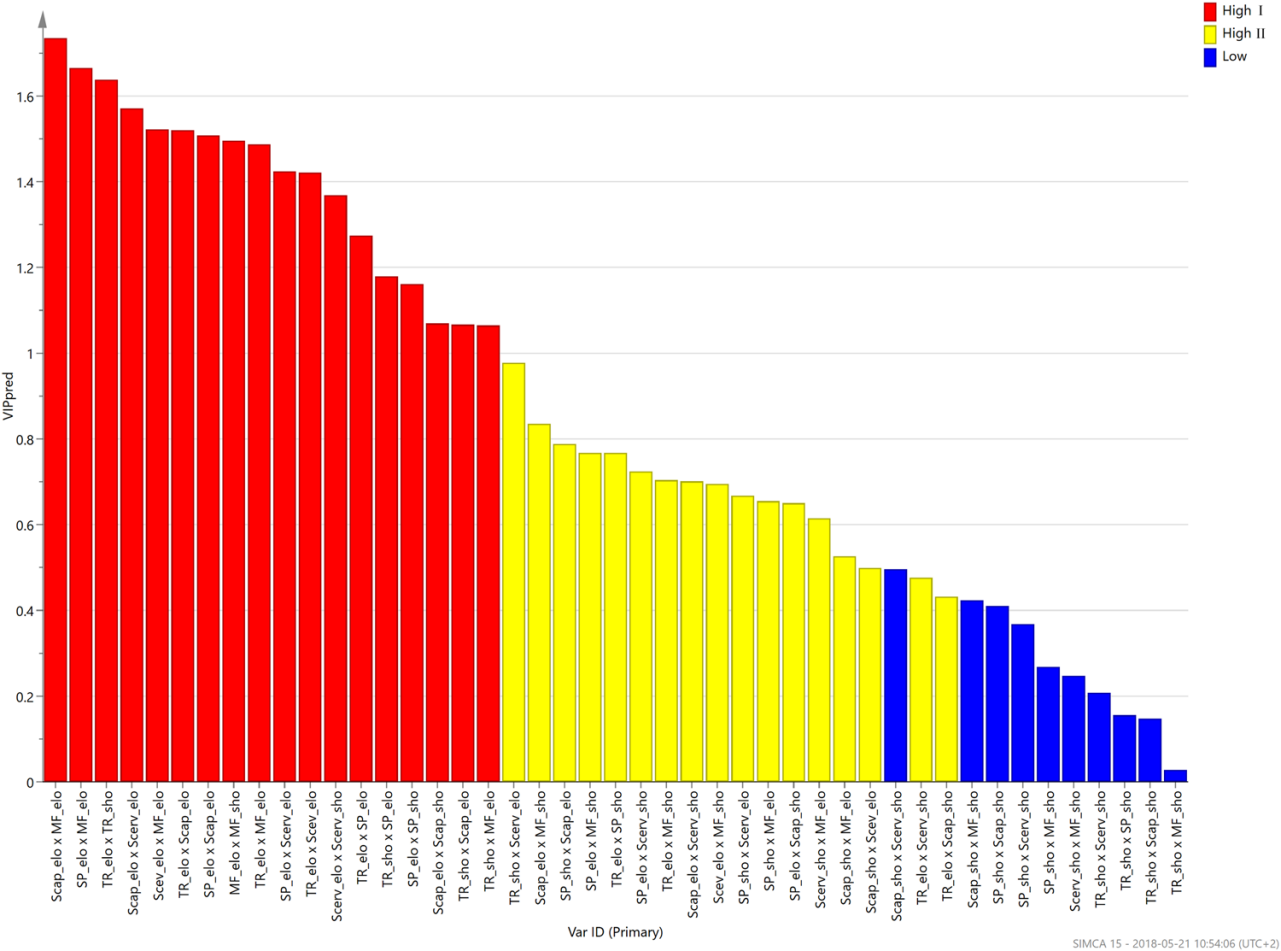
Variable influence for projection (VIP) plots. Variable influence for projection (VIP) plot for two-way dorsal muscle interactions and maximum voluntary isometric contraction as response. The interactions are sorted by their importance in the predictive OPLS component. The most important interactions, with values above 1, are seen in red.

**Figure 8:**
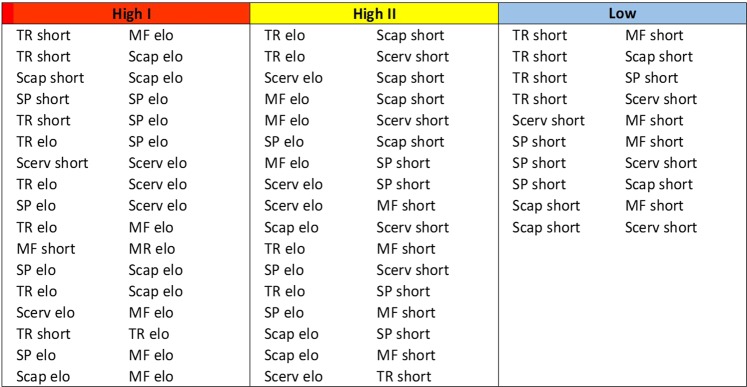
Variable influence of projection (VIP). The most important variables for interaction between the five dorsal muscles deformation were investigated using variable influence of projection (VIP). Two-way interaction between the dorsal neck muscles showing the differences in deformation (elongation; elo, shortening; short) during different percent of maximal voluntary contraction (High I; 60–80% MVC, High II; 40% MVC, low; 10–20% MVC,). The five dorsal neck muscles are; Trapezius (TR), Splenius capitis (SP), Semispinalis capitis (Scap), Semispinalis cervicis (Scerv), and Multifidus (MF). Seventeen of the variables were positively related to High I (red; 60–80% MVC) and High II (yellow; 40% MVC) maximal voluntary contraction. For Low MVC was 10 variables positively correlated (blue; 10 and 20% MVC).

The corresponding loading scatter plot for the predictive and orthogonal components (Fig. 6) revealed the importance of the two-way dorsal muscle interactions. The interactions could be broadly categorized into three classes; High I, strong interactions activated during high MVC; High II, interactions activated during high MVC but less pronounced; Low, weaker interactions activated during low MVC. The variable importance for the projection (VIP) plot, (Figs 7 and 8), shows the interactions in a sorted manner, from high to low importance.

OPLS was also used to investigate the relationship between the dorsal muscle two-way interactions and the sex and age of the participants. The same cross-validation procedure as for MVC was employed to assess the connection between the interactions and sex/age. Weak models, with negative Q2Y values, were obtained in both cases. This suggests these responses cannot be predicted from the dorsal muscle interactions and no relationships exist.

### Reliability among the three trials

Deformation measurements showed strong reliability for all five muscles together (ICC: 0.79; 95% CI: 0.71–0.85), and fair to good reliability for the five muscles separately (ICC: 0.43–0.74; Table 3).

**Table 3:** Reliability coefficients (Intraclass Correlational Coefficients (ICC)) for muscle deformation area and force for each muscle separately and the five muscles together.

Muscle	ICC (95% CI)
Trapezius	0.51 (0.30–0.61)
Splenius Capitis	0.43 (0.20–0.60)
Semispinals Capitis	0.74 (0.64–0.82)
Semiespinalis Cervicis	0.58 (0.41–0.71)
Multifidus	0.66 (0.53–0.76)
Sum of 5 muscles	0.79 (0.71–0.85)

## Discussion

The study findings support our hypothesis that cervical muscle deformation versus time curves is a better method for evaluating ultrasound speckle tracking analysis than calculating the average RMS^17^. Our recent study^17^ analyzing ultrasound speckle tracking using average RMS values of deformation curves^19,20^ showed a weak relationship between RMS and force (R^2^ = 0.03–0.18). In contrast, the present study showed that 16 of 20 individuals demonstrated a subject-specific strong to very strong linear relationship (9 individuals) or non-linear relationship (7 individuals) between the percentage of MVC and muscle deformation when the five neck muscles were summed together. The linear relationship was strong and the non-linear relationship was moderate for normalized deformation values (in the entire group). The multivariate OPLS analyses showed high explained model variance (R^2^Y = 0.61) and predictive variance (Q^2^Y = 0.55) for the interaction between neck muscles. The neck muscles were shortened at low force (10% and 20% MVC); however, at higher force (40% and 60% MVC), the two-way interactions were characterized by one elongated muscle and one shortened neck muscle. Lastly, the majority of the interactions for the 80% MVC level involved elongation of both muscles. The multivariate analyses revealed subject-specific muscle interactions between the muscles during isometric MVC test, with different combinations and deformations of the five neck muscles during the test. The reliability of the three tests used to measure total deformation was substantial when the five muscles were summed together but was lower for each muscle separately. However, at MVC ≥ 40%, the ultrasound measurement revealed deformation in the muscles according to movement of the cervical spine (visually seen in the ultrasound image). Thus, the results (≥40% MVC) was not only related to isometric neck muscle function.

The individuals displayed very different amounts of muscle deformation. At the same %MVC, some individuals displayed high deformation and others displayed low deformation. This variability was consistent with findings in other studies examining muscle activation (including EMG and ultrasound), indicating high individual variability in muscle activation patterns, rather than stereotypical patterns in pain and non-pain conditions^6,7,12,15,21–24^. EMG measures the neuromuscular activation of muscle fibres, and ultrasonography measures the mechanical deformation within the muscle. The two methods measure different physiological activities, but both are essential for movement. Albeit different, both these methods have indicated high variability among individuals. EMG studies of muscle activity after experimentally induced pain revealed subject-specific muscle activation in six low back muscles^21^, in the calf muscle^22^ and in the neck muscles; indeed, no two of the eight participants showed the same muscle adaption pattern after painful stimulus in the splenius capitis muscle^6^. Previous ultrasound investigations of neck muscles revealed an individual interplay between ventral^15^ and dorsal^12^ neck muscles in individuals without neck pain. Men typically have larger muscle sizes and greater strength compared to women^25^. Moreover, ultrasound investigation of dorsal neck muscles revealed that men had much greater variation in muscle deformation than women^12^. However, using OPLS modelling, neither sex nor age were found to have any relation to dorsal muscle deformation in this study. The outcome may have been affected by each individual’s muscle strength, endurance, routine physical activity, and training level. Although most participants showed variability between muscles and between trials, some individuals did not. Lower movement variability can be an indicator of skilled performance^26^, with better coordination between muscles, and it may explain why some individuals presented a very strong relationship between force and %MVC, in addition to high reliability among the three trials. However, this hypothesis is speculative, because we did not investigate the activity and training level of participants in the present study. Despite the variability between individuals, the multivariate analyses revealed that at low force levels of the neck extensors (10% and 20% MVC), both deep and superficial neck muscles were shortened. While the relationship between muscle deformation and muscle activation still needs to be interpreted with some caution, it seems unlikely that the observed muscle shortening at low force was the result of longitudinal pressure but rather it was the result of muscle contraction in these healthy individuals.

We found that approximately half of the participants showed linear (9 of 16) and the other half showed non-linear (7 of 16) relationships between the %MVC and total muscle deformation. In a recent study, a curvilinear relationship was reported between an isometric test of 5–90% MVC and superficial EMG signals in the upper and lower dorsal neck muscles^27^. Approximately one third of those curves displayed a quadratic shape, one third displayed a piecewise linear pattern, with a breakpoint at about 25% MVC, and one third fit both quadratic and linear patterns. In a similar study by Woods *et al*., both linear and nonlinear relationships were reported^28^ with the authors speculating that the non-linear relationship between force and EMG may be related to the proportions of the different muscle fibre types. Muscles with mixed type I and II fibers showed a non-linear relationship with force, and muscles with more uniform fibre composition showed a linear relationship with force. EMG studies have also shown a complex activation pattern in the dorsal neck muscles, including subject-specific preferred activation of the splenius as a neck extensor or flexor^7,23,24^ and subject-specific activation strategies during a reflexive task^24^. Studies on the morphometry of neck muscles have investigated the physiological cross-sectional area (PCSA) of muscles, muscle mass, density, pennation angle, and fascial length; they also defined the principal line of action and the presence of aponeurotic and tendon attachments^29^. The PCSA, together with myosin heavy chain and fiber type distributions are the main factors that contribute to the muscle length-force relationship^30^. Kamibayashi and Richmond^29^ showed a complex neck muscle architecture with substantial differences between individuals; in a small female, the PCSA of the trapezius muscle might be larger in one part, but only 70% of the male PCSA in other parts. The splenius capitis has been described as a very complicated muscle, quilted by internal tendons and exhibiting different architectures in different parts of the muscle^29^. In the semispinalis cervicis, the synaptic input is distributed non-uniformly to different fascicles, which may be due to the different mechanical functions of individual muscle fibers^31^. This complex pattern and different morphometry in neck muscles within and between individuals may explain the great variation in deformation values and the individual force and deformation relationships presented in this study.

**Figure 9:**
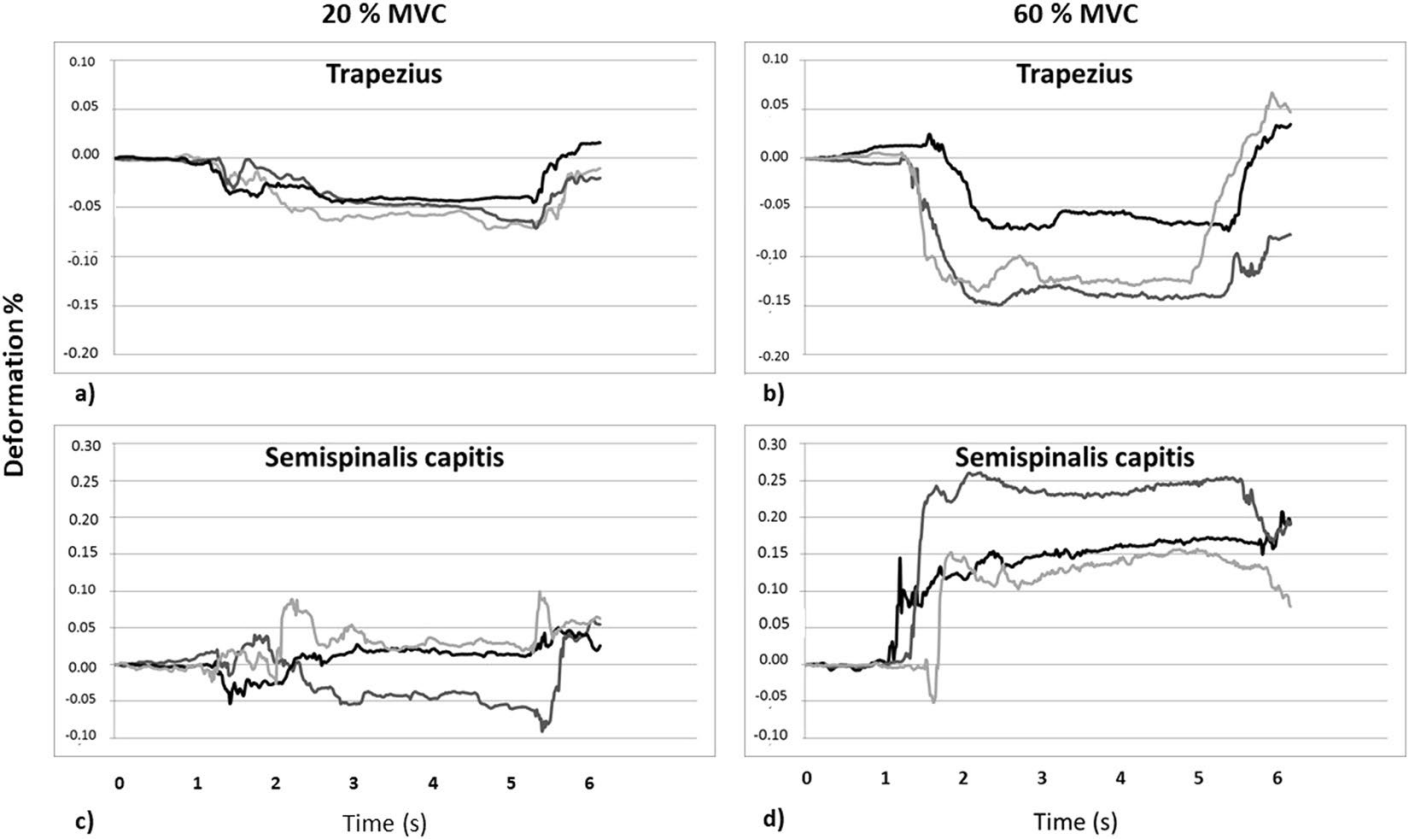
Muscle deformation diagrams. These diagrams illustrate muscle deformations over time for the trapezius (**a**,**b**) and semispinalis capitis (**c**,**d**) muscles in one individual. Three tests were performed at (left; **a**,**c**) 20% MVC and (right; **b**,**d**) 60% MVC. Each line (black, gray, and light gray) represents the change in deformation (%) observed in the ROI on the ultrasound image, in one muscle for one test. Deformations below zero (negative values) represent muscle shortening, and deformations above zero (positive values) represent muscle elongation. The total area under the curve (sum of negative and positive areas) represents the total muscle deformation during the test. When the line crosses the 0% line, the muscle switches from shortening to elongation, or vice versa.

The motor control system is complex and variable. Humans never seem to replicate a movement exactly^26^. The redundancy of neck muscles allows variation^1^ and some muscles can be activated, when other muscles rest during a repeated test. That may explain, to some extent, the mediocre reliability of the ICC values found in the present study for the five neck muscles analysed separately and the large differences between the three trials in some individuals. Figure 9 shows that neck muscle deformation (splenius capitis, 20% MVC) could change substantially between the three tests; it could be elongated in one test and shorted in the next test. These variations in neck muscle deformation between repeated movements may protect joints and muscles from overuse, because the tissues and structures are not repeatedly loaded in the exact same way. A previous ultrasound investigation of muscle deformations in the biceps brachii found that the inter-subject variability was higher than the intra-subject variability^11^. Moreover, during the isometric MVC trials in the present study, in some individuals, the deformation values showed shortening in one or more of the five neck muscles, while other muscle(s) showed elongation. Deformation within the muscles in the longitudinal direction implicates thickening in the vertical direction. Consequently, the vertical pressure from an active muscle will cause the displacement of passive surrounding muscles, which will be seen as elongations in the longitudinal direction^14^. This interaction implies that speckle-tracking analyses from five adjacent muscles will present both active and passive deformation values. However, before clear conclusions can be drawn, further tests of muscle activity in all five neck muscles taken concurrently with ultrasound speckle tracking analyses need to be performed.

In the present study, more elongation was detected in the deep muscles during higher %MVC forces; at 60 and 80% MVCs, all five neck muscles were more elongated than shortened during the trials. The trials were performed in an upright position with each participant’s thorax secured anteriorly and posteriorly. However, the cervical spine was moveable, and higher force may have changed the position of the neck to a more forward position. This movement was observed in the visual inspection of the ultrasound images. In trials from 40% to 80% MVC, the cervical spine moved in a forward direction. It is generally assumed that synergistic muscles such as the deep ventral neck muscles will physically support the cervical spine^32^ during forceful contractions. However, high force and possibly activation of multiple neck muscles might have contributed to changing the postural position of the cervical spine during the higher force trials in this study. Thus, we cannot confidently state that muscle deformation observed at high force was due to muscle (eccentric or concentric) contraction. At low force, 10–20% MVC, both deep and superficial muscle were shortened in the present test and the cervical spine had not moved on visual inspection of the ultrasound images. Thus, ultrasound speckle tracking analyses may detect muscle contraction in neck muscles at low force.

Previous studies have shown that MVC could change muscle length. At an optimal joint angle, the gastrocnemius muscle was shortened by as much as 35–47%^33,34^. However, to our knowledge, no study has investigated the limit of muscle shortening in neck muscles. In leg, arm, and abdominal muscles, contractions less than 30% MVC were associated with relatively large changes in muscle length, but at higher levels of contraction, the muscle changed relatively little^35^. Lopata *et al*.^11^ reported relatively large deformations at 30% MVC in the biceps brachii muscle, and they suggested that force and muscle deformation were not directly proportional. Interpretation of the present results is challenging, due to the possible forward movement of the cervical spine and changes in head and neck positions that may occurred in contractions ≥40% MVC.

When the five neck muscles were analysed separately, only the splenius capitis showed a linear relationship between deformation and force. For the trapezius and semispinalis capitis muscles, the curves peaked at 60–80% MVC; and for the two deepest muscles, the semispinalis cervicis and multifidus that are proposed to physically support the cervical lordosis, the curves peaked at 40% MVC. The test-retest reliability within one day showed only fair to good reliability for each muscle. The suboptimal reliability may have been due to the high variability in human movements, the redundancy of neck muscles^1,23,29,31^ and the potential of combining different muscles during repeated test. However, when the five neck muscles were summed together, we observed substantial reliability (ICC: 0.79; 95% CI: 0.71–0.85), similar to that reported in an EMG study, where the reliability between tests varied from 0.79 to 0.83 (mean ICCs, within-day testing)^27^.

Ultrasound measurement of muscle deformation have been investigated successfully against force measurements; to track local tissue deformation for the biceps bracchi muscle^11^. In that setting, the measured force during voluntary contraction showed good resemblance with the deformation curves. Ultrasound measurements of muscle deformation can show active and passive muscle functions. Eccentric contractions imply active muscle elongation, and concentric contractions imply active muscle contraction. Passive muscle deformation is typically observed as an elongation of the muscle, due to muscle fatigue or stretching in response to pressure from surrounding tissues. Thus, it was not possible to draw any conclusions about active or passive muscle functions in the present study. However, speckle-tracking analyses showed strong to very strong relationships between muscle deformation and the %MVC; this result indicated that this method could detect changes in the dorsal neck muscles related to force. The visual inspection of the ultrasound video sequences showed no movement of the cervical spine in a forward direction in the lower %MVCs. Thus, the results indicated that ultrasound with speckle tracking analyses may have application at lower levels of force (<40% MVC) to detect and measure neck muscle deformations also recommended in other studies^11,35^. At higher %MVC levels, the deformations may be more related to changes in other muscles and/or changes in head and neck positions. Four individuals showed only moderate linear or non-linear relationships between muscle deformation and %MVC. All were men, but the sample size was too small to allow sub-groups analyses between genders. Most of the subjects (n = 16) showed an individual subject-specific strong relationship between %MVC and muscle deformation.

The results indicated that calculation of muscle deformation vs time should be used in ultrasound speckle tracking analyses. Using this method, the result showed a subject-specific performed mechanical neck muscle pattern with different solutions for deformation of the five dorsal neck muscles during isometric neck extension testing from 10% to 80% MVC. For most individuals, the relationship between deformation and force was moderate to very strong, with substantial test-rest reliability, when all five muscles were summed together. In summary, measures of muscle deformation appear to be most useful at lower levels of muscle force (<40% MVC) in dorsal neck muscles. Further studies now need to investigate a larger number of force increments within this range (for example 5%, 10%, 15%, 20%, 25%, 30%, 35% MVC) that also includes participants with neck pain.

## Methods

This study utilised ultrasound images and corresponding force data from our previous study^17^.

### Participants

This study included 20 healthy subjects, 12 males and 8 females, mean age 24 years (SD 5.3), recruited from the university and the general community. Participants were included if they were right handed, and reported no history of neck pain or headaches for which they had sought intervention, and demonstrated no signs of physical dysfunction (decreased range of movement and/or pain) during an examination of the cervical spine. An experienced physiotherapist specialised in musculoskeletal disorders, examined all participants. Participants were excluded if they reported present or past neck problems, including generalized myalgia, rheumatologic disease, or neurological disease.

The study was approved by the Institutional Research Ethics Committee at the University of Queensland, and it was conducted in accordance with the Declaration of Helsinki. Written informed consent was obtained from all participants after they were given information about the experiment.

### Test procedure

Ultrasound imaging was recorded during the performance of isometric cervical extensor muscle contractions. Muscle contractions were guided with a custom-made isometric dynamometer^36^. The selected contraction intensities were of 10%, 20%, 40%, 60%, and 80% of maximal voluntary contraction (MVC). The participants stood during the experiment, with their thorax secured anteriorly and posteriorly. The head and neck were in an upright neutral position, and the arms were relaxed. Just below the participant’s external occipital protuberance, an adjustable dynamometer lever arm was positioned. The dynamometer axis was in line with the transverse process of C7. For each trial, an application pad resisted the subjects’ cervical extension effort and the resultant torque (referred to as force in this manuscript) was recorded at the dynamometer axis. The force recordings were synchronized with the ultrasound recordings. This dynamometry method has shown to be highly reliable for measuring cervical extension force^36^.

The participants were led through a standard warm up procedure that comprised five submaximal isometric neck extension contractions before the experiment started. Then, three MVCs were performed. The participants were verbally instructed to increase their neck extension effort from rest to maximal contraction over 3 s, then to hold the contraction level for 2 s, before returning to rest. Between each trial, a 2 min break was allowed to minimize fatigue. Visual feedback was provided by showing the participant a target line on a visual display and the MVC trials were conducted until the participant achieved two maximal torque values within 5% of one another or they performed a maximum of four trials. The greatest force achieved in the 3–4 MVCs was used to calculate the submaximal target force values for the subsequent experiments.

The experimental tasks were performed at least 3 min after the final MVC. For the submaximal test, subjects performed five isometric neck extension trials at 10%, 20%, 40%, 60%, and 80% of MVC force. Each trial lasted 6 s, and the order of contraction intensity was randomized between subjects. Subjects performed three trails at each force intensity level, and between contractions, they rested either 30 s, for intensities below 50% MVC, or 1 min for intensities above 50% MVC. The subjects were instructed to ramp up the cervical extension force, from rest to the target level of force, which was displayed on the feedback screen. The target force was held for approximately 3 s, and then participants were instructed to reduce their force to rest.

Force and ultrasound images were recorded simultaneously throughout all muscle contraction trials and an electronic synchronization signal (a trigger) was elicited when the muscle was deemed to be at rest, just prior to the beginning of each contraction, and then again, when the participant was holding the desired force level.

### Ultrasound measurement

**Figure 10:**
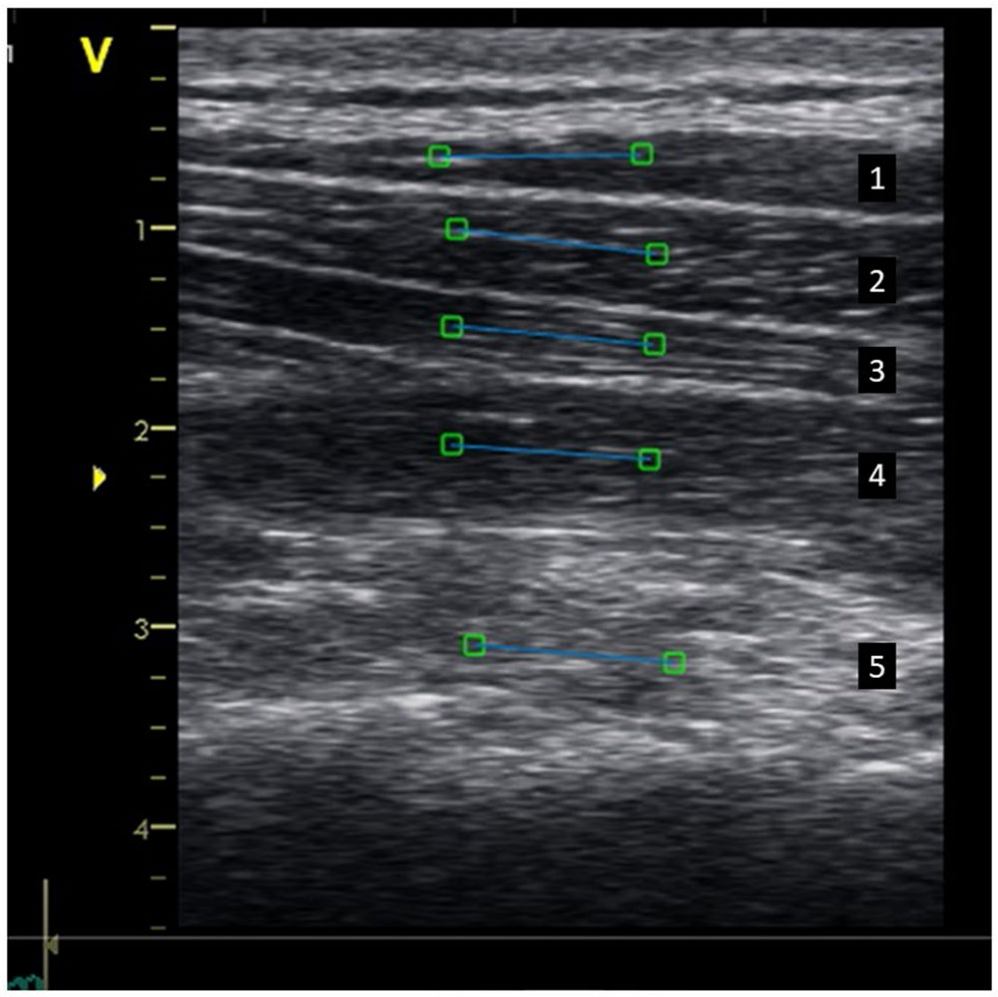
Ultrasound image of the dorsal neck muscles with Region of Interest (ROIs). Muscles are arranged in layers (from top to bottom): (1) trapezius, (2) splenius, (3) semispinalis capitis, (4) semispinalis cervicis, and (5) multifidus/rotatores. Five regions of interest (ROIs; each indicated as a blue line with a square on each end) were selected in each muscle for post-process speckle tracking analysis.

The dorsal neck muscles were evaluated using a B-mode, two-dimensional (2-D) ultrasound Vivid-i scanner (GE Healthcare, Horten, Norway) and a 12.0 MHz linear transducer (38 mm footprint) with a frame rate of 50 frames/s. In each recording, five dorsal neck muscles (upper trapezius, splenius capitis, semispinalis capitis, semispinalis cervicis, and multifidus/rotatores) were captured (Fig. 10). The transducer was placed on the right side of the neck, at the level of the spinous process at the C4 level, in a transverse orientation to identify the articular process, lamina, and the different layers of the dorsal neck muscles. Next, the transducer was rotated 90 degrees, to a longitudinal position that provided an optimal image of the five dorsal neck muscles. Minimal force was applied to the transducer, in such way that a clear ultrasound image was seen but without compression of the neck muscles. The position of the ultrasound transducer was marked on the subject’s skin with a pen. Hence, the position of the transducer could be standardized between the tests. A single examiner (AP) identified the correct position and held the ultrasound transducer in all experiments for all participants.

Ultrasound of muscle results in reflection of soundwaves, which serve as acoustic markers because they form a unique speckle pattern. A gray-scale image on muscles is composed of several bright speckles, due to small irregularities in the muscles. This acoustic marker, speckle pattern, is unique and remains relatively stable over time, and can therefore be followed frame-by-frame in a sequence of images^37^. To track the unique speckle pattern in the neck muscles, a region of interest (ROI) was manually selected in each of the five muscles (Fig. 10).

A research software for skeletal muscles was developed^10^ for detection, visualisation and quantification of skeletal muscle deformation. The speckle tracking method was implemented in MATLAB, currently compiled in MATLAB 2018b (9.5.0.1033004, The Mathworks Inc, Natick, MA, USA) using the optical flow tracking algorithm developed by Kanade, Lucas and Tomasi^38,39^. The software tracks the unique speckle pattern within the ROI, where each ROI is defined by a number of points equivalent to every other pixel in the ROI. This gives around 80 points for a ROI size of 15 mm given the current video resolution of 486 × 418 pixles. The points are used in a least squares approach^40^ for finding the ROI end points of each tracked image frame. The tracking also incorporates forward-backward error^41^ where each tracked point is tracked back to the previous frame and the distance between the tracked point and the original point is given as the error. For the current study, we used a forward-backward error of 2 for the elimination of unreliably tracked points.

One ROI (15 mm) was manually placed in the middle of each muscle in the first frame of the video sequence (Fig. 10), oriented longitudinal to the muscle fibres. When the muscle changes in length, the tracked ROI also changes, and the speckle pattern can be followed, frame by frame, throughout the ultrasound images. The tracking software then calculates the deformation of each ROI in each frame sequentially and compare the length with the initial resting ROI frame using Langrangian formula. Lagrangian strain is the cumulated deformation, divided by the initial length and shows deformation (elongation or shortening) relative to the constant baseline length. The displacements of each ROI and frame were summed over all the frames in the video to obtain a cumulative sum, which provided quantitative information about muscle behaviour during the trials.

Muscle deformation (elongation and shortening) was defined as a change in the ROI length, and it was calculated as the percentage change (% deformation) from the original length of the muscle at rest (first frame). Thus, each ROI provided information about the local muscle dynamics. The results are presented as total deformation (both shortening and elongation), muscle elongation, and muscle shortening.

### Data analyses

To evaluate muscle deformation, the areas under the muscle deformation vs. time curves were calculated (Fig. 9). As a basis for evaluating the area (Eq. 1), the trapezoidal rule was used; *A* as the area under the curve, *t* was the time between samples, and *y*_*n*_ was the present ROI position at sample point *n*. To handle intersections with the 0% line, we modified the equation. We used linear interpolation with adjusted *t*-values at intersections with the 0% line to estimate additional sample points. Thus, the negative area (values < 0%) and the positive area (values > 0%) could be separated. To quantify the deformation during the test, area under the curve was calculated by summing the areas of successive trapezoids for the total duration of the whole test.

1 $$A=\frac{t}{2}({{\rm{y}}}_{1}+2{{\rm{y}}}_{2}+{2}_{{\rm{y}}3}+\mathrm{..}+2{{\rm{y}}}_{{\rm{n}}-2}+2{{\rm{y}}}_{{\rm{n}}-1}+{{\rm{y}}}_{{\rm{n}}})$$

The mean deformation value of the three repetitions was used in the analyses of 10%, 20%, 40%, 60%, and 80% MVC. Neck muscle strength and endurance differs between men and women and between subjects of the same sex^25,42^. Therefore, mean deformation values were normalized to the 100% MVC value for each subject. These normalized values allowed us to compare deformations between different individuals.

### Statistical analysis

Statistical analyses were performed with SPSS software (IBM SPSS, Statistics for windows, Version 24.0, Armonk, NY). A linear fit was performed for each subject to examine the relationship between the mean deformation areas, based on raw data from the three tests, and the force intensity (%MVC). The goodness of the linear fit (measured with adjusted R2 values) was used to evaluate the strength of the relationship and were categorized as follows: weak, 0.1–0.3; moderate, 0.31–0.6; strong, 0.61–0.9; and very strong relationship, >0.9. Analyses were performed for all five neck muscles together and for each of the five neck muscles separately, based on the mean values of the three tests. The areas under the curves for the total deformation, the shortening, and the elongation are presented as the mean ± SD. Normalized deformation area values (deformation values normalized to each subject’s 100% MVC) were analysed and compared between individuals. The reliability of force and deformation measures (for each muscle separately and for all five muscles summed together) over the three trials were evaluated and expressed as intraclass correlational coefficients (ICC), and inter-rater agreement was categorized by the ICC value, as follows: poor <0.40, fair 0.40–0.60, good 0.60–0.74 and strong >0.75^43^. A one-way repeated analysis of variance (ANOVA) with Bonferroni correction was used to evaluate how the change in deformation area for each of the five neck muscles (trapezius, splenius, semispinalis capitis, semispinalis cervicis, and multifidus/rotatores) was related to the %MVC. When the sphericity assumption (Maucley test) was violated, a Geisser–Greenhouse correction was used. The level of significance was set to p < 0.05.

### Multivariate data analyses

To further investigate the deformation areas and specifically the interaction between the five dorsal muscle layers, a multivariate data analysis approach was employed. Two-way interaction terms were generated for all combinations of the shortening and elongation areas of the five muscle layers, 45 terms in total. Interaction terms were generated as the element-wise multiplication of a and b

$${i}_{ab}=\frac{a-{\bar{x}}_{a}}{{s}_{a}}^\circ \frac{b-{\bar{x}}_{b}}{{s}_{b}}$$

where ${\bar{x}}_{a}$ and ${\bar{x}}_{b}$ are variable means and *S*_*a*_ and *S*_*b*_ are the standard deviations. Prior to calculating the interaction terms, the areas for each of the three trials for the same participant and MVC were normalized with the recorded elapsed time for the corresponding trial. The average MVC value was subsequently calculated from the three trials. For some participants, only two trials were registered, and consequently two values were used for calculating the average value in these particular cases.

The 45 averaged and normalized interaction terms between the five dorsal muscles and their shortening and elongation areas were assembled in a matrix **X**, which was subjected to multivariate data analyses. Prior to the analysis, the variables were logarithmically transformed with base 10 followed by centring and scaling to unit variance. Principal component analysis (PCA) was used to compress the matrix into a few components^18^. PCA scores were used to explore the multidimensional interaction data in 2D and 3D scatter plots to find groupings, trends or possible outliers. Orthogonal partial least squares^44^ (OPLS) regression was applied as a supervised technique between the interactions, and MVC. OPLS finds and removes variation in **X** that is orthogonal to the response, **y**. It then finds a predictive component between the correlated variation and the response. The strength of the OPLS model was determined by its R^2^Y and Q^2^Y values, the model explained and the predictive model explained variances, and the related root mean squared error of cross-validation (RMSECV). A R^2^Y or Q^2^Y of 1.0 represents best possible explained variance, while the RMSECV should be as low as possible. RMSECV is expressed in units of the modelled response, i.e. in this case the MVC. The cross-validation was carried out in a leave-one-subject-out manner, which resulted in all trials (from MVC 10% to 80%) for same participant were excluded and predicted at the same time. Additionally, OPLS predictive scores were used to assess the visual difference between the five MVC levels. Also, OPLS loadings were studied to interpret differences between the five MVC levels in regard to their respective interaction pattern among the dorsal muscle layers. Furthermore, the relationship between the dorsal muscle interactions and sex and age of the participants was investigated with OPLS.

## Data Availability

The datasets analysed during the current study are available from the corresponding author on reasonable request.
